# Chronic pain and unrecognized grief: epistemic barriers to personal and social recognition

**DOI:** 10.1007/s11019-026-10333-7

**Published:** 2026-03-09

**Authors:** Christopher Jude McCarroll, Ying-Tung Lin, Dominik Koesling, Claudia Bozzaro

**Affiliations:** 1 Institute of Philosophy of Mind and Cognition, National Yang Ming Chiao Tung University, Taipei, Taiwan; 2 Institute of Ethics, History and Theory of Medicine, University of Münster, Münster, Germany

**Keywords:** Chronic pain, Grief, Loss, Depression, Epistemic barriers

## Abstract

What is it to grieve? What is the nature of grief? An intuitive and straightforward answer to these questions, one that will be familiar to all of us, is that grief is an emotional reaction to the death of a close loved one. Grief is intimately connected to bereavement. However, grief can arise in situations beyond the death of a significant other and is revealed to be a much more complex and heterogeneous experience. People can grieve over all sorts of losses. What makes our response to these losses one of grief? We can understand all these losses as involving a loss of life possibilities, which impacts one’s practical identity and who one takes oneself to be. Importantly, a close examination of the phenomenology of chronic pain helps illuminate the ways in which it also involves the kind of losses that we can grieve over. The losses involved in experiences of chronic pain impact one’s practical identity in ways that can lead to grief. This chronic pain grief remains largely unrecognized, however. We outline four epistemic barriers to recognizing the grief involved in experiences of chronic pain.

Poor Sisyphus grief

I am not ready for your heaviness

Cemented to my body

Look closely and you will see

Almost everyone carrying bags

Of cement on their shoulders

That’s why it takes courage

To get out of bed in the morning

And climb into the day

—Edward Hirsch^1^

“Physical” pain is an experience, and one in which grief, depression, isolation can provide its very texture and intensity.

—Drew Leder (2016)

## Introduction

What is it to grieve? What is the nature of grief? An intuitive and straightforward answer to these questions, one that will be familiar to all of us, is that grief is an emotional reaction to the death of a close loved one. Grief, on this view, is a sibling of bereavement and mourning. We are embedded in webs of interpersonal relations, some significant, some less so. We often suffer the loss of these relationships during the course of our lives, and we respond emotionally to this loss. When we suffer a bereavement and lose someone significant, such as a parent or a partner, that is when we experience the throes of grief. This view is not wrong. Grief is often, and perhaps typically, a response to a bereavement. However, the way in which grief manifests is much more complex than this. Indeed, the nature of grief itself is not simply feeling a particular emotion. Grief is a process that ebbs and flows over time; it has psychological and physical, as well as socio-cultural and normative aspects in the sense that there can be a prescribed temporal structure to grief or the emotions it evokes, as well as requiring us to examine and perhaps rethink our commitments and values (e.g., Cholbi 2019; Hughes 2022; Goldhill 2017; O’Connor 2022; Markovic 2024). Grief is something that fundamentally changes us and our experiences of the world (Attig 2011).

However, grief can arise in situations beyond the death of a significant other and is revealed to be a much more complex and heterogeneous experience, as can be seen by considering the following instances. People can grieve over all sorts of losses. In some cases, people feel grief for individuals they never personally knew: experiences of grief can extend to public reactions to the passing of famous figures or cultural icons. Further, experiences of grief are not limited to human relationships. People often grieve the loss of their pets (Archer & Winchester 1994). Grief also occurs for non-death losses. For instance, individuals may mourn the break-up of a romantic relationship (Solomon 2004). Parents of children with severe mental health conditions can also experience grief (Williams-Wengerd & Solheim 2021), as can parents dealing with the emotional challenges when their adult children move out of the family home (Schultz & Harris 2011). Indeed, the losses involved in grief may not be limited to individuals but can extend to the material world. Many people report grief over the destruction of the natural environment (Fernandez Velasco 2025; Fernandez Velasco & Richardson 2026), or the loss of homes and historically significant structures to natural disasters or fires (Roberts 1992; Varga & Gallagher 2020). Grief has also been reported for losses that are abstract. For example, people have grieved over the loss of employment, the loss of personal freedom, or the loss of political possibilities (Ferraro et al. 2011; Richardson et al. 2021; López Ríos et al. 2024).

The way in which grief can arise is complex. What we want to do in this paper is add a further dimension of complexity to this story. But it will be an element of complexity that is illuminating. We suggest that grief is intimately linked to, and involved in, experiences of chronic pain. Indeed, highlighting this connection between grief and chronic pain helps shed light on, and enables us to think better about, these important aspects of human suffering. A number of theorists have begun to explore the relations between grief and chronic illness (Byrne 2022; Cole & Ratcliffe 2022) as well as between grief and chronic pain (Dysvik & Furnes 2010; Furnes & Dysvik 2010; Garciandía Imaz & Rozo Reyes 2019). However, despite the intuitive (and perhaps emerging theoretical) link between psychological and physical pain, this is a topic that remains undertheorized.

Indeed, as we show, there are (at least) four interrelated epistemic barriers to recognizing chronic pain as involving experiences of grieving: 1) as we intimated above, there is the misconception of grief as always being a response to bereavement and the loss of a significant other. 2) This invisibility is also frequently a part of chronic pain experiences, where the object of loss in chronic pain might not be visible. This invisibility of chronic pain relates to the assumed visibility of bodily pain. The standard interpretation of pain is that it is a biological phenomenon, but this neglects the psychological and social aspects of chronic pain experience. 3) The third epistemic barrier relates to differing conceptions of the temporal structure of grief and chronic pain. Typical grief as a response to bereavement is thought to unfold in a relatively linear fashion with a clear starting point, whereas the temporal unfolding of experiences of chronic pain is much more fluid and dynamic. This encourages people to think that chronic pain does not involve grief. 4) Experiences of chronic pain are often misunderstood as involving depression rather than grief. We suggest that the losses of chronic pain are most often the losses of grief, not depression. While chronic pain may involve or lead to depression, we suggest that experiences of chronic pain are crucially related to grief; in fact, highlighting this connection might enable therapeutic interventions that could prevent the chronic pain sufferer from entering depression.

What we want to do in this paper is to dismantle these epistemic barriers and examine the connection between chronic pain and grief in a new light. In order to do this, we first outline a way of thinking about the nature of grieving that can help us make sense of the various ways in which we can experience grief and the varied losses that we can grieve over (Sect. "Grief: A Loss of Life Possibilities"). We then explore experiences of chronic pain. A close examination of the phenomenology of chronic pain helps illuminate the ways in which it also involves the kind of losses that we can grieve over (Sect. "Chronic Pain as a Complex Experience of Losses"). Our main claim is that there is such a thing as chronic pain grief. Here we also situate our account in relation to the extant literature that has gestured at the connection between chronic pain and grief. We also highlight the ways in which thinking about chronic pain in terms of grief makes sense by thinking of them as both involving unchosen transformative experiences: they are experiences that involve losses of things we deeply value and hence change us in profound ways. Finally, in Sect. "The Invisibility of Chronic Pain Grief", we outline the reasons why chronic pain grief often goes unrecognized. We describe in more detail the epistemic barriers to acknowledging chronic pain grief. By making this phenomenon more visible we hope that this might afford new possibilities of potential therapeutic interventions.

## Grief: a loss of life possibilities

How can we explain the diversity of grief and get a better understanding of the nature of grieving? One important point to first emphasize is that rather than thinking of it as a discrete state, event, or psychological episode (e.g., a feeling, a perception, a judgement), which can be understood and captured at a particular moment in time, grief is best understood as a process (Goldie 2011). Grief is “a complex pattern of activity and passivity, inner and outer, which unfolds over time” (Goldie 2011, p. 119).^2^ On this processual view, grief is a process that unfolds over longer-term temporal durations and is composed of distinct emotional episodes (e.g., anger, sadness, yearning, shock) at different temporal points (Ratcliffe & Byrne 2021). So, grief is a process. But how can we explain the seemingly disparate ways in which grief can occur? What makes these diverse instances of grief precisely instances of the same emotional process—*grief*?

One way to make sense of this complexity of grief, and to unify experiences of grief, is to appeal to the intentional structure of emotions in general, and of grief in particular. It is widely accepted that many affective experiences are intentional in nature—that is, they are directed toward or concern specific objects or states of affairs (Scarantino & de Sousa 2021). At first blush, the thing that grief seems to be directed towards is the loss of the person. But then, as we saw, the actual losses involved in grief may vary significantly and may include many non-death losses. The precise nature of the loss that grief is about remains elusive. One way of understanding the losses of grief and making them more transparent is to appeal to a distinction between two aspects of the intentional structure of emotions. When we think of the objects of emotions, we can make a distinction between the *concrete* (or *particular*) object and the *formal* object of the emotion (de Sousa 1987; Teroni 2007; Cholbi 2021; Ratcliffe et al. 2022). Concrete (particular) objects refer to the specific entities that emotions are directed toward. The formal object of an emotion is often understood as an evaluative property that renders the concrete (particular) object an appropriate or fitting target of the emotion (Lyons 1980; cf. Teroni 2007):

any *X* that I can have emotion *E about* is a particular object of *E*, whereas the formal object of *E* is the property which I implicitly ascribe to *X* by virtue of having *E about X*. (Scarantino & de Sousa 2021)

To take a standard example, consider the case of fear. We can experience fear for many different things, and fear different things at different times. One person’s fear may be directed towards spiders, another person’s fear may be about snakes, while yet others may experience no such emotion when presented with these creatures. These are different instances of fear. Yet what makes them all instances of fear nonetheless?^3^ It is often thought that despite their differences, these concrete objects share a formal object. In the case of fear, this formal object is typically identified as *danger* or *threat* (Teroni 2007; Ratcliffe et al. 2022). We fear *things* (concrete/particular objects) that we evaluate as *dangerous* or *threatening* (this is their formal object): “Generally speaking, then, there is a content to all fear, which could be generalised as ‘the dangerous’. It is this generalised content to all fears that is called the formal object of fear” (Lyons 1980, p. 99).

In grief too, as we saw, we find a diversity of concrete (particular) objects. While the range is extensive, appealing to grief’s formal object may help to unify these varied experiences. Several candidates have been proposed for grief’s formal object (see Cholbi 2021). One particularly promising account characterizes the formal object of grief as the *loss of life possibilities* (Ratcliffe et al. 2022; cf. Varga & Gallagher 2020). Here, the loss concerns not just the person or thing itself, but the erosion of possibilities that were essential to the agent’s practical life—those embedded in habits, projects, relationships, and ongoing commitments.

Understanding the formal object of grief in this way seems to reveal a tension with grief’s processual nature. If grief is a process that unspools over time, rather than a state that is instantiated at a distinct moment in time, Ratcliffe and colleagues (2022) suggest that the traditional distinction between the concrete and formal objects of emotion might not adequately capture experiences of grief. One way to usefully apply the notion of a formal object to experiences of grief is, however, to think of the distinction between concrete and formal objects as a part-whole relation: “The process [of grief] as a whole engages with a loss of life possibilities, while constituent experiences relate to more specific aspects of this loss, which have varying degrees of concreteness” (Ratcliffe et al. 2022, p. 15). In this way, the insight that grief is a process can be combined with the idea that it involves a formal object, which can be understood as a loss of life possibilities. Moreover, this focus on the loss of life possibilities affords an understanding of the way in which grief may ebb and flow, and may eventually diminish over time. The formal object of grief may not attach to a single concrete object during the grieving process but rather “different aspects of a wider-ranging loss of possibilities are experienced as such at different times” (Ratcliffe et al. 2022, p. 3).

Furthermore, thinking of grief as involving the loss of possibilities affords the recognition of the past, present, and future-oriented aspects of grief. For example, the concrete losses involved in grief, which are evaluated as involving a loss of life possibilities, need not be confined to past-oriented or historically anchored losses. Ratcliffe and Richardson (2023) emphasize that grief often arises from the loss of *future* possibilities that were integral to one’s life. Their phenomenological study of involuntary childlessness shows that what is grieved is not the loss of a concrete past object, but the foreclosure of an anticipated future—children imagined, roles envisioned, and identities tied to parenthood. Accordingly, grief is not just a reaction to something that has happened, but a response to the collapse of a life trajectory and to the impossibility of becoming the person one expected to be: “it is a *core characteristic* of the experience of disorientation in grief that the grieving person can no longer meaningfully press ahead into a *specific futural self*” (Mehmel 2023, p. 991). In this sense, the objects of grief do not need to correspond to an “actual” loss, in the sense that you had something and now you no longer have it, but they can correspond to any aspect of one’s lifeworld that one evaluates as bringing about a loss of life possibilities.

To deepen this account and make this notion of a loss of life possibilities somewhat more tractable, we can connect it to an account of grief that understands it as involving changes to one’s practical identity (Cholbi 2021). Understood in this way, the losses of possibilities that are the formal objects of grief involve disruptions to one’s practical identity (McCarroll & Yan 2024). Practical identity is “a description under which you value yourself, a description under which you find your life to be worth living and your actions to be worth undertaking” (Korsgaard 1996, p. 101). One’s practical identity encompasses the set of commitments, values, and concerns, which one uses to guide the decisions one makes and the actions one takes. It is important to emphasize that practical identity is a relational notion. These values and concerns are not simply isolated to the individual, but are often dependent on relations to others. In this way, our practical identities involve “roles and relationships, citizenship, memberships in ethnic or religious groups, causes, vocations, professions, and offices” (Korsgaard 1996, p. 20). Losing life possibilities in grief involves experiencing and negotiating a fractured sense of who one is. When we lose someone or something integral to our practical identity, we experience a loss of life possibilities and with it an unravelling in the fabric of the self (Kramer 2025; Cholbi 2025).

Viewing grieving through this lens of a loss of life possibilities affords a way of understanding chronic pain as an experience that can involve grief. A description of the phenomenology of chronic pain reveals that it involves precisely the kinds of losses of life possibilities that are the (formal) objects of grief.

## Chronic pain as a complex experience of losses

Recently, scholars from various fields have recognized the interrelations between grief and experiences of long-term illness and chronic pain. Cole and Ratcliffe (2022), from a phenomenological and clinical perspective, situate illness-related grief within non-death losses. They propose that the common structure linking bereavement and illness is the loss of life possibilities: illness can be understood through the same intentional and temporal structures that characterize bereavement grief. On their account, illness-related grief’s formal object is the loss of life possibilities, specifically the erosion of practical horizons, roles, and projects that constitute a person’s sense of self. Illness involves a collapse of these possibilities and a reorganization of bodily and temporal experience: the body becomes conspicuous, time thickens, and the future narrows. Byrne’s (2022) case study of myalgic encephalomyelitis/chronic fatigue syndrome (CFS/ME) demonstrates that chronic illness can elicit a pervasive grief that is easily misclassified as depression. Patients experience a continual confrontation with loss—of identity, bodily trust, social recognition, and future possibilities—but these losses are rarely validated because prevailing medical and psychological frameworks privilege depressive symptomatology. However, both Cole and Ratcliffe (2022) and Byrne (2022) observed that grief in chronic illness is both ambiguous and disenfranchised (Boss 1999; Doka 2002): the losses are ongoing rather than terminal, and the absence of culturally sanctioned rituals or public acknowledgment leaves sufferers without the social scaffolding needed for meaning-making.

Furnes and Dysvik (Dysvik and Furnes 2010; Furnes and Dysvik 2010) explicitly connect bereavement with grief accompanying chronic pain, proposing a unified theoretical and therapeutic framework. They suggest that grief, loss, and chronic pain involve “relearning the world” and “adaptation”, which consist of a continuous movement between experiences like despair—hope, loss—relearning, meaning disruption—meaning reconstruction, bodily discomfort—bodily reintegration. Based on this framework, their intervention model integrates cognitive-behavioral reflection, expressive writing, interpersonal dialogue, and therapeutic alliance, emphasizing that recovery involves re-authoring one’s life narrative and reintegrating bodily and existential meaning.

While existing studies have explored the connections between grief, chronic illness, and chronic pain, what remains missing is a fine-grained phenomenological account of the specific kinds of losses in chronic pain that constitute grief. The phenomenological analysis that follows addresses this gap by mapping these losses onto the framework of grief as a loss of life possibilities.

The first loss that goes ahead with the upcoming of a recurring and never-ending pain is the loss of the ‘normal’ or habitual experience of one’s own lived body. Following the assumption of body-phenomenology as established by Merleau-Ponty, the body is not merely a physical or biological entity. It is also the lived experience of a person (Merleau-Ponty 2011). The notion of embodiment is predicated on the premise that the human body exerts a profound influence on our perception of the world and our interaction with it. The prevailing paradigm of human bodily experience is typified by a perpetual sense of bodily self-awareness, despite the physical presence of the body being largely overlooked. However, individuals are often not typically cognizant of their bodies while engaged in routine daily activities. The human body is characterized by its presence in the mode of absence (Leder 1990). It is evident that this experience undergoes a radical transformation in the presence of pain. The body in pain suddenly commands our attention. Having pain in the stomach, the head, or the back makes us aware that we have a stomach, a head, or a back. Long-lasting and intense pain focuses attention on the body and thus prevents habitual patterns of being in the world through the body. With time, pain can become the dominant theme in life, as Patient Petra Andresen describes^4^:

It starts with you talking primarily about your pain. You realize that you have nothing else on your mind. Then you withdraw because you can no longer do much. You do your work. The weekend takes place in bed. This is followed by increasing inactivity, which of course also clearly leads to muscular atrophy and this is actually a classic progression of a pain patient withdrawing completely.

Sometimes the pain can be so intense that it eclipses or overrides all other perceptions and thus has a completely overwhelming, even totalizing (Grüny 2004) and alienating effect (Svenaeus 2015). Additionally, chronic pain is often further accompanied by comorbidities including *stress*, *sleep* or *anxiety disorders*, *fatigue, depression*. Such disruptions can lead to the obstruction of how one habitually experiences and engages with one’s surroundings. As pointed out by Cole and Ratcliffe (2022), it is important to

distinguish aspects of grief that are principally concerned with the body from others that involve loss of access to something else due to bodily changes. […] losses change not only how we experience our bodies, but also how we experience and relate to our surroundings (p. 160).

Chronic pain has been shown to modify not only the physical experience, but also the experience of time (Fuchs 2002; 2020), leading to a loss of the known way to deal with time. Usually, individuals tend to act as competent administrators of their time, meticulously planning, utilising and investing their time. People relate to the future through plans, wishes and hopes. They look back on the past through memories. The present is often not experienced at all since the movement of time, its passing by, is continuous and not explicitly perceived. The experience of time is like the experience of the body, fugacious in the sense that people are usually not aware of the passing of time. However, the more intense and persistent the pain, the more inevitable the confrontation with time, as both the prospect of the future and the review of the past are lost. You are trapped in the pain and with it in a “negative present” that will not go away.

The word “chronic” comes from the Greek word chronos: “time”. In medical terms, this is because chronic pain is defined by how long it lasts. But this aspect can also be deepened in relation to the experience of pain. Chronic pain is not just pain that lasts (a little) longer. Because of this time component, it is something completely different: a complex experience of suffering. This suffering is mainly due to the fact that chronicity means that there is no more pain-free future. While the thought of a pain-free future is justified in the case of acute, that is short-term pain, and gives the sufferer perspective and hope, the chronic pain sufferer is often condemned for life to remain in a painful present from which no pain-free future is possible. The future of the chronic pain sufferer is blocked. Emmanuel Bäckryd describes this change as follows: “the person in chronic pain hence becomes aware of the self as a temporal being, i.e., as a being immersed in a temporal world in which the past haunts you with grim memories and the future looms as a dark place one does not want to visit” (Bäckryd 2023, p. 4332). The time trajectory of chronic pain is further challenging because its intensity is never constant. There may be interruptions or periods when the pain subsides, but when it will subside and, above all, when it will occur again, is usually uncertain for the patient (Grüny 2004, esp. pp. 167–176). Therefore, any attempt to maintain a structured life in the face of this condition is doomed to failure, as Patient Monika Roth’s testimony illustrates:

Well, I can’t just go about my life like this or muddle along or say: ‘Gosh, tomorrow we’ll do this and then we’ll do that.’ [...] It’s spontaneous, even more spontaneous, because then I can say: I can go on this hike right now [...] Or: I'm in so much pain right now [...] that I can’t walk that much...

In many cases, people with chronic pain have limited or no ability to participate in everyday activities. What others consider normal becomes impossible for them because of their pain. Chronic pain therefore marks a rupture with normality in many respects. This is particularly evident in terms of social participation, as chronic pain can make activities such as walking or getting around effortlessly impossible, is often associated with the loss of a job, and changes the social status and relations of those affected within their families and among their friends. However, it is also evident in terms of existential issues such as one’s own self-image, one’s idea of a good life and life goals, which people had hoped to achieve, but in the course of chronic pain have to realize that these plans cannot be adhered to, or at least not in the same way (cf. Kieselbach et al. 2023, p. 118). Patient Andrea Müller comments on this:

I actually had to [give up] one thing after another of the things that were important to me, that I enjoyed, yes - they didn’t work anymore. So I had to stop doing sport, playing guitar, making music and all those things.

To make matters worse, chronic pain remains invisible compared to many other conditions, which raises doubts about the authenticity of pain complaints, often resulting in a perceived loss of credibility – especially since those who are not affected usually only know acute pain from their own experience, which is generally much less restrictive in its effects. As a result, the needs of people with chronic pain are often *ignored* or *misrecognized* (cf. Koesling & Bozzaro, 2022, p. 564–565). On the contrary, many patients report the mistrust they experience from people who cannot imagine what it is like to be in constant pain.

The loss of tasks, the forced withdrawal from work and other activities leave many people with chronic pain with a sense of uselessness and a fear of being a burden to others. It is not uncommon for people affected by chronic pain to report feelings of loneliness, which can even lead them to feel *disconnected from their fellow human beings*, or even to feel as if they live in a different *world apart from others*, what Patient Anna Wagner describes in the following way:

One aspect [...] that probably applies to every type of chronic illness is the experience that healthy people and sick people live in different worlds. [...] And that’s nobody’s fault or nobody wants it that way, it’s just the nature of the situation - [...] Because you can’t share certain experiences in this way, you can’t expect it either, because healthy people simply live in a completely different rhythm. And the chronically ill person or - in this case - the chronic pain sufferer - they don’t die, but they don’t get better either. [...] - And that doesn’t stop....

Isolation, loss of life plans and life goals frequently result in those affected questioning the value of living a life with chronic pain.^5^ This is particularly true in cases where the search for an explanation of pain remains unanswered and links with questions of self-blame related to one’s suffering, as is the case with patient Christa Schumacher who asks:

Why actually? Why do I have all this? Sometimes it’s not so easy to understand. [...] What have I done to have to live the way I do? I would also like to go to a greenfield site.

A pain that has no meaning, no reasonable explanation, and no solution can lead to a loss of meaning, joy, and hope. This is particularly important because *meaning*, which can be thought of as an understanding of the (possible) “relationships among things, events, and relationships” (Baumeister 1991. p.15), is often seen as a crucial aspect of well-being (e.g., Crisp 2025; Steger 2017).

Of course, not all types of the losses discussed here occur at the same time, nor are they experienced by everyone with chronic pain. However, this brief overview of the various loss experiences that can accompany chronic pain illustrates why individuals with chronic pain may also be grieving. They experience the same kinds of losses that are grieved over in bereavement. These losses are losses that involve a disruption to one’s practical identity. They are losses of life possibilities. This can involve the loss of actual concrete things, such as the normal functioning of their bodies, their job and friendships, but also objects in a more abstract sense, such as meaningful life projects that can no longer be realized, meaning and self-esteem. All these losses experienced during chronic pain can be understood as involving losses of life possibilities. They involve losses of the relations, roles, careers, pastimes, and hobbies, that go to make up an individual’s practical identity. It is over these types of losses, which can manifest in chronic pain just as in bereavement, that the individual grieves. Experiences of chronic pain can result in grief that unfolds unrecognized.

One way of drawing out the connection between chronic pain and grief is to think of them as involving unchosen transformative experiences. These kinds of experiences are experiences that shape the self in profound ways, epistemically and personally or existentially, but that were not chosen by the individual (Markovic 2022).^6^ Grief, illness, and accident are examples of unchosen transformative experiences (Carel et al. 2016; Markovic 2022). They result in alterations to one’s identity but they come unbidden. It is important to emphasize that grief is only one example of an unchosen transformative experience. Not all such experiences involve grief or result in grief. The question of what makes something an instance of grief is not the same as what makes something an unchosen transformative experience (Markovic 2024). Grief involves more than just an adjustment to a loss. However, as Jelena Markovic explains, for an unchosen transformative experience to count as grief

The loss must be valuable—this can be captured by the notion, for instance, of an investment of one’s practical identity…, importance to one’s flourishing…, inclusion in one’s orientation to the future… or the presence of a system of possibilities associated with the lost object…(Markovic 2024, p. 254)^7^

Importantly, the losses that are manifest in experiences of chronic pain are precisely those that are valuable: they impact one’s practical identity, they are important to one’s flourishing, they impact how one envisions one’s future, and they are embedded in systems of life possibilities. Chronic pain sufferers need to renegotiate their identities in the light of these profound losses.^8^

Chronic pain grief is an unchosen transformative experience. However, the experience of grief in chronic pain is something that is often not transparent and remains opaque, both to the people suffering chronic pain themselves and to observers of this suffering more generally. In the next section we outline a number of epistemic barriers that inhibit us from seeing chronic pain grief.

## The invisibility of chronic pain grief

This highlighted dimension of loss associated with chronic pain is less frequently discussed within the context of chronic pain research, but has been the subject of some discussion in the literature from grief research. One way in which this has been done is to suggest that grief itself manifests in sensations of physical pain, or experiences of grief can cause physical pain including chronic pain. For example, in an analysis of interviews with bereaved people, Pearce and Komaromy (2022) acknowledge the essential role of the body in the grieving process and highlight that the “bodily pain of grief described by the participants demonstrates the significance of the materiality of the body in how grief is experienced” (2022, p. 406). So, grief can be one *cause* of physical pain. This causal connection between grief and chronic pain was also shown in a study where school nurses observed pain in adolescents:

One SN [school nurse] talked about a girl who had a stomach ache for three years, even though she was examined at the hospital several times without result. No one linked her pain to having lost a close family member. However, when she finally joined a grief group, the stomach ache disappeared. (Høie et al. 2017, p. 4)

In this way of thinking chronic pain would be a *symptom* of grief, or a way in which grief is expressed. Pain becomes a “disguised metaphor” for a different problem that has been somaticized (Geniusas 2020, p. 180). This type of connection between chronic pain and grief is an important one. However, even here there is a degree of invisibility to the relationship between chronic pain and grief. The standard way of thinking about grief in these models is, again, as the response to a bereavement. Grief and subsequent pain occur, in this view, when we lose a significant other. Yet as we have been emphasizing, grief can also occur for other kinds of losses, real or imagined. This aspect of grief remains relatively opaque and if the resulting grief experience manifests in or contributes to chronic pain, then this connection is less likely to be visible.

There are, we propose, at least four relevant, yet not adequately recognized and addressed, epistemic barriers that cause this. It is important to stress that we propose these barriers to enable further dialogue and discussion without suggesting that these account for all the relevant factors. Nonetheless, as we see it, these are important elements that cloud the visibility of chronic pain grief. We now address these epistemic barriers in detail:

### (A) The misconception of grief and the invisibility of the losses underlying grief in chronic pain

The first epistemic barrier concerns the way grief is usually conceptualized. As reviewed in Sect. "Grief: A Loss of Life Possibilities", grief can be understood as a loss of life possibilities, a framework that helps unify its diverse forms. While scholars have identified grief in various kinds of non-death losses, including chronic pain (Dysvik & Furnes 2010; Furnes & Dysvik 2010; Garciandía Imaz & Rozo Reyes 2019), the standard understanding of grief still centers on bereavement—the loss of a loved one. This narrow conception remains prevalent and contributes to the limited recognition of grief in other contexts. As a result, even though some patients may privately acknowledge grief in chronic pain, such recognition is neither widespread nor clinically acknowledged. This misconception can lead to failures in distinguishing healthy grief from pathological grief or depression in diagnosis and treatment (see below).

Doka (1999, 2002, 2006) introduces the concept of “disenfranchised grief” to refer to grief that occurs when a loss is not socially and publicly recognized or supported, leaving those in grief isolated without communal validation. Disenfranchised grief may occur for several reasons: when the relationship with the deceased is unrecognized, when the loss itself is unacknowledged, when the griever is socially marginalized (e.g., individuals with disabilities), when the loss carries stigma (e.g., suicide), or when the mode of grieving is socially invalidated. For patients suffering from chronic pain, the invisibility of loss is the primary reason their grief is often disenfranchised, but other factors also contribute. Chronic pain carries social stigma, and the way individuals grieve the losses differs markedly from the grief typically associated with bereavement.

### (B) The (assumed) visibility of bodily pain

The second epistemic barrier relates to the fact that a predominant interpretation of pain – on an individual, institutional and societal level – is that of a biological phenomenon. This historical primacy, which continues to exert an influence in the present, is reflected in the current definition of the International Association for the Study of Pain (IASP) defining pain as “[a]n unpleasant sensory and emotional experience associated with, or resembling that associated with, actual or potential tissue damage” (Raja et al. 2020, p. 1977).^9^ This emphasis on tissue damage relates to a focus on the “visible”, the objective counterpart of the subjective sensory and emotional experience. The most prominent image that people have in mind when thinking about pain is that of a visible wound. Indeed, many experiences of acute pain are related to visible tissue damage. However, this is not the case in chronic pain. Often, a biological cause cannot be identified and in many cases of chronic pain, there is no organic correlation that can be made visible, even through technical imaging such as X-rays or MRIs.

This emphasis on tissue damage has further led to the psychological, social, and existential dimensions of pain being overlooked or subordinated, which is particularly significant in cases of chronic pain. Chronic pain may be particularly complex because it may involve multiple interacting causes and effects (Coninx 2024).^10^ Within the field of specialized pain medicine, the complex nature of chronic pain has been recognized and is expressed with the so-called *bio-psycho-social model of pain*. This model expresses this complexity of chronic pain and makes clear that in addition to biological aspects, psychological and social ones are also an integral part of chronic pain. However, this comprehensive view and understanding of chronic pain is still not widely known outside of specialized circles, and even though it may be recognized by clinicians there may be challenges to consistently implementing it (Epstein & Borrell-Carrio 2005), and we might need a variety of pedagogical resources in order to explain it to different groups (Dong & Bäckryd 2023). Indeed, in some instances, even in healthcare practice and chronic pain management there is a tendency to emphasize the biomedical aspects of pain (Sheridan et al. 2025), whereby “psychosocial factors are often assigned secondary status and viewed largely as reactions to pain” (Meints & Edwards 2018, p. 168). As a result, most people do not properly recognize, either fully or even in part, the psychological and social aspects as integral parts of the suffering of chronic pain. Moreover, even if people do recognize these psychological and social aspects, given the typical understanding of grief as a bereavement response that we examined above, there is less of an awareness of the ways in which non-death losses can be part of the *psycho-social* sphere that is inherent to chronic pain experience.

### (C) Different assumptions concerning time

A third relevant epistemic barrier concerns time. Since the most prominent experience of grief is grieving an irreversible loss of a loved person or thing, grief is typically conceptualized as a (relatively linear) process that starts with the experienced loss, is undergone and will ultimately reach its conclusion after various stages (Kübler-Ross & Kessler 2005). In this context, there are often rituals that accompany the grieving process, for example, funerals, which provide the bereaved with an opportunity to bid farewell to their loved one. However, in the context of chronic pain grief, this dynamic is notably different. As Ratcliffe notes on temporal trajectory:

Taking an unexpected and sudden bereavement as our exemplar, we might think of grief as a temporally extended engagement with the implications of something that has already happened. However, pain and discomfort are often experienced over a lengthy period. Furthermore, those with chronic progressive conditions do not merely comprehend and adapt to something that has already happened — there is continuing change in one’s condition, often involving considerable uncertainty and the anticipation of further losses. (Cole & Ratcliffe 2022, p. 165)

The experience of chronic pain is not characterized by a single loss, but rather by repeated “small” losses – often interspersed with intermittent moments of hope that the loss may not be permanent, only to be followed by subsequent disillusionment. It can also be difficult for someone affected by chronic pain to identify exactly when they experienced a loss, because some losses are rather subtle and gradual. For example, the loss of friendships may not happen suddenly; rather, friends may withdraw over an extended period of time. Consequently, this type of grief must be regarded as an unending process. Grief in the context of chronic pain therefore encompasses an indefinite, ongoing, cyclical and evolving time frame. This can make it difficult for the affected individuals themselves to become aware that they are grieving as well as for society more broadly.

### (D) The misidentification of grief in chronic pain as depression

The fourth and last epistemic barrier to be addressed here relates to the conflation of grief and depression. Both grief and depression share features that make them difficult to distinguish from one another. Because of these similarities, when there is no obvious trigger such as bereavement or a clearly identifiable loss, grief is often misidentified as depression and treated as such. Scholars have argued that rates of comorbid depression are inflated in cases involving non-death grief, for example, in chronic illnesses such as Chronic Fatigue Syndrome/Myalgic Encephalomyelitis (CFS/ME) (Byrne 2022). We suggest that the difficulty in differentiating grief from depression constitutes an epistemic barrier that prevents chronic pain patients and others from recognizing grief in their experiences.

Nonetheless, some distinctions between bereavement and depression have been identified. For instance, significantly more patients suffering from depression exhibit symptoms of worthlessness and hopelessness compared to those experiencing grief (Clayton et al. 1974). Ratcliffe (2018) further differentiates grief from depression by arguing that grief involves the loss of a system of possibilities—one can no longer pursue particular hopes tied to the deceased or to the loss—whereas depression entails the loss of the capacity to hope or to find significance at all, and is in this sense more fundamental. Moreover, grief “involves dynamic perspective-shifting” and “a sustained ability to relate to and feel connected with other people,” both of which are notably absent in depression (Ratcliffe 2019). It should be noted, however, that chronic pain patients may also experience feelings of worthlessness or hopelessness, or lack the interpersonal connection that Ratcliffe associates with major depression. Different forms of non-death grief may exhibit distinct characteristics depending on the nature of the loss, sharing varying degrees of similarity with depression. Determining which features reliably distinguish grief from depression in chronic pain experiences will require further empirical investigation.

## Concluding remarks

The standard understanding of grief is that it is a response to the death of a loved one. Yet both everyday experience and scholarly theorizing recognize that the way in which we can experience grief is more complicated than this. Grief not only follows from the death of a closed loved one, but can also occur in a variety of other contexts of loss. One such context is the experience of suffering from chronic pain. As we have demonstrated, this experience has the capacity to effect a fundamental change in an individual’s life: it becomes unfeasible to engage in a multitude of activities in the same way that were previously part of everyday life, or, in some cases, are rendered completely impossible due to persistent or recurring pain. For instance, individuals may encounter circumstances that render them unable to continue working in their chosen profession, their mobility may be restricted, and they may be unable to maintain the same level of social interaction with friends, gradually leading to drifting apart from their social circles and social isolation (e.g., Koesling & Bozzaro 2021; Breivik et al. 2006). Those afflicted with chronic pain, and their families, may experience a radical transformation in their lives as a consequence of the condition (cf. Kieselbach et al. 2025). This is particularly evident in the modification of personal life plans. This transition, which can be conceptualized as a diminution in opportunities, can be profoundly challenging, and this complex reality must be recognized – recognized without resorting to direct pathologisation.

As we have shown, the relation between grief and chronic pain is complex. In some cases, grief after a bereavement might lead to chronic pain, and in this sense grief might be a contributing causal factor in the experience of chronic pain. However, the kinds of losses that individuals who are suffering chronic pain experience can themselves be the proper objects of grief. In this way the connection between grief and chronic pain is deeper. In some instances, chronic pain can be a form of grieving. Importantly, we have also demonstrated that epistemological barriers hinder the recognition of grief in chronic pain. Some of these barriers concern common assumptions about what ‘typical’ grief and ‘typical’ pain are. These assumptions are one-sided and undermine the complexities of these experiences. Others are related to misconceptions about the different time processes associated with ‘typical’ grief and chronic pain, and the misidentification of grief in chronic pain as depression.

Improving support for people suffering from chronic pain requires acknowledging their grief. Therefore, we encourage the implementation of proper psychological support focusing on grief needs in the management of chronic pain patients. Perhaps some insights can be gained from palliative care and applied to chronic pain management. In the literature on palliative care there has been some work looking at grief reactions in both the terminally ill as well as their caregivers (Bilić et al. 2022; Coelho & Barbossa 2017); this work has started to examine the way the ways in which (terminal) illness can result in the kinds of losses that are grieved over (McCarroll & Yan 2024), and sets out guidelines for bereavement support (Coelho et al. 2025).^11^ At the same time, we need to increase understanding of the heterogeneity of pain and grief within the scientific community and the general public.^12^

## Footnotes

1 Quoted in Stroebe (2018).

2 According to Goldie (2011), what unifies the diverse elements of grief—which can include thoughts, feelings, imaginings, actions, and memories—is the narrative that the grieving person constructs. For Goldie, narratives can provide powerful explanatory, revelatory, and expressive functions that simple causal explanations cannot. Some authors have challenged Goldie’s focus on narrative as the unifying element of the grieving process (Ratcliffe & Byrne 2022). What is important for our purposes is not the narrative aspect of Goldie’s account, but the processual nature of grief.

3 There is a debate about whether the formal object of an emotion is what individuates it as an instance of a particular emotion type. See, for example, Teroni (2007) for discussion.

4 All patients’ quotations are taken from the homepage “krankheitserfahrungen.de”. This is the licensed website of the Database Project for Individual Patient Experiences Germany (DIPEx Germany), which is based at the Institute for Social Medicine and Epidemiology at the Brandenburg Medical School Theodor Fontane. The names of patients are on the webpage but are pseudonyms to protect their anonymity. The quotations were translated by Claudia Bozzaro.

5 This touches on the existential questions surrounding meaning, the loss of meaning, and the search for meaning in the context of chronic pain, highlighting the profound challenges that individuals experiencing this condition face. For further exploration into this domain, see, for instance, van Hout et al. (2023), Kieselbach et al. (2023) and Wade & Shantall (2003).

6 There may be resonances here with Karl Jaspers’ (1932/1970) notion of *boundary situations*, which can involve existential and personal transformations. Moreover, boundary situations may be important for thinking about spiritual care in a secular setting (Gutschmidt & Frick 2025), which may be important for therapeutic interventions to alleviate suffering in chronic pain grief (see Sect. "Concluding Remarks" below).

7 Markovic situates the sense in which the loss is valuable in terms of certain influential accounts of grief. Value in relation to an investment in one’s practical identity relates to the work of Cholbi (2019; 2021), as importance to one’s flourishing relates to Nussbaum (2001), as inclusion in one’s orientation to the future relates to Mehmel (2023), and in terms of being related to a system of possibilities connects to the work of Ratcliffe (e.g., 2019).

8 These losses can also permeate the lives of caregivers too in ways that manifest in grief. A caregiver may experience losses of intimacy and relationships, losses of freedom and time for their own pastimes, hobbies, or work (e.g., McCarroll & Yan 2024; see Sect. "Concluding Remarks" below).

9 For philosophical engagement with and defence of the IASP definition of pain, see Aydede (2017), and for a more critical, phenomenological approach, see Mickiewicz (2025).

10 For discussion on the complexity of pain, see, for instance, Corns (2020).

11 Another literature that might be helpful to mine is the work done on “total pain” (e.g., Saunders & Baines 1983), which has attempted to outline ways of alleviating and managing elements of pain such as existential suffering (Kissane 2012) and instilling meaning in the lives of patients (Fisher et al. 2023).

12 We leave it to future work to fully investigate the kind of therapeutic interventions that might be afforded when one recognizes chronic pain grief.
